# Arginine/Nanocellulose Membranes for Carbon Capture Applications

**DOI:** 10.3390/nano9060877

**Published:** 2019-06-10

**Authors:** Davide Venturi, Alexander Chrysanthou, Benjamin Dhuiège, Karim Missoum, Marco Giacinti Baschetti

**Affiliations:** 1 Department of Civil, Chemical, Environmental and Material Engineering (DICAM), Alma Mater Studiorum, University of Bologna, Via Terracini, 28, 40131 Bologna, Italy; davide.venturi16@unibo.it (D.V.); A.Chrysanthou-15@student.lboro.ac.uk (A.C.); 2 INOFIB, Rue de la papeterie, 461, 38402 St-Martin-d’Hères, CEDEX, France; benjamin.dhuiege@inofib.com (B.D.); karim.missoum@inofib.com (K.M.)

**Keywords:** CO_2_ separation, facilitated transport, nanocellulose, amino acid, gas separation membranes

## Abstract

The present study investigates the influence of the addition of l-arginine to a matrix of carboxymethylated nanofibrillated cellulose (CMC-NFC), with the aim of fabricating a mobile carrier facilitated transport membrane for the separation of CO_2_. Self-standing films were prepared by casting an aqueous suspension containing different amounts of amino acid (15–30–45 wt.%) and CMC-NFC. The permeation properties were assessed in humid conditions (70–98% relative humidity (RH)) at 35 °C for CO_2_ and N_2_ separately and compared with that of the non-loaded nanocellulose films. Both permeability and ideal selectivity appeared to be improved by the addition of l-arginine, especially when high amino-acid loadings were considered. A seven-fold increment in carbon dioxide permeability was observed between pure CMC-NFC and the 45 wt.% blend (from 29 to 220 Barrer at 94% RH), also paired to a significant increase of ideal selectivity (from 56 to 185). Interestingly, while improving the separation performance, water sorption was not substantially affected by the addition of amino acid, thus confirming that the increased permeability was not related simply to membrane swelling. Overall, the addition of aminated mobile carriers appeared to provide enhanced performances, advancing the state of the art for nanocellulose-based gas separation membranes.

## 1. Introduction

One of today’s major global concerns is represented by the excess of environmental greenhouse gases (GHGs), such as CO_2_, in the terrestrial atmosphere [1]. Global warming issues, which are strongly related to these gases, are indeed now recognized by a vast majority of law-makers, and severe actions must be taken in order to minimize emissions and the overall impact of high concentrations of carbon dioxide and other GHGs [2].

Within this frame of thought, technologies such as carbon capture and storage (CCS) appear to play a significant role for short- to medium-term goals in the reduction of anthropogenic CO_2_, while a zero-emission path for energy production is built. A CCS approach is based on the removal of CO_2_ from flue gases of large-scale energy productions and other industrial activities, and the sequestration of the compressed gas in underground deposits [3,4]. 

To perform this operation, efficient separation techniques must be adopted in order to guarantee an acceptable degree of purity, while maintaining low costs. Traditionally, carbon dioxide is separated from light gases, such as N_2_, CH_4_, CO, and H_2_, by means of chemical absorption, constantly regenerating the solvent. This methodology is currently quite optimized, but it brings with itself several disadvantages, such as relatively low energy efficiency, difficulty in operability, and the use of harmful organic solvents [5]. Gas separation membranes represent an alternative solution that is gaining an increasing amount of interest in the chemical and process industry, thanks to the absence of additional fluids, reduced energy needs, and absence of moving parts [5,6,7,8]. 

Membranes commonly utilized by the industry rely purely on a solution–diffusion mechanism to act as a molecular sieve substantially based on the kinetic diameter and condensability of the various molecules [9]. This mechanism, however, tends to suffer from an inherent trade-off between permeability and selectivity, which limits the separation performance of the materials [10,11]. 

A number of strategies were developed over the years to overcome in some way this limitation, using, for example, mixed matrix membranes [12,13,14] or polymer and block copolymers with high CO_2_ affinity [15,16,17,18]. Recently, a higher focus was given to facilitated transport membranes (FTM); in this class of materials, the diffusion of a specific molecule (in this case, carbon dioxide) is driven not only by a solution–diffusion mechanism, but also by a reaction of the gas species with functional groups embedded in the matrix, commonly named “carriers” [19,20,21]. The permeation of a specific gas, or an ensemble of gases with similar chemical characteristics is, therefore, facilitated as they can diffuse both as free molecules or via carrier-mediated transport [22]. This way, since only the desired molecule transport is increased, both overall permeability and selectivity toward other species are enhanced, possibly overcoming the previously described limitations of the solution–diffusion mechanism [23,24]. 

In the case of carbon dioxide separation, amine moieties represent a fairly reasonable choice which were exploited in several works [25,26,27,28,29]. The mechanism itself, which regulates the interaction between aminated molecules and CO_2_, is not completely understood, even though two main reactions are commonly considered to occur within the matrix [29,30]. When unhindered amines are present, carbon dioxide tends to form a carbamate ion through a zwitterion mechanism, a pathway originally described by Caplow [31] and presented in Equations (1) and (2):

(1) $$CO_{2}+R-NH_{2}⇌R-NH_{2}^{+}-COO^{-},$$

(2) $$R-NH_{2}^{+}-COO^{-}+R-NH_{2}⇌R-NH-COO^{-}+R-NH_{3}^{+}.$$

A single molecule of the target gas interacts with two amine groups in order to be facilitated in its diffusion. If a hindered amine is present, a second mechanism is preferred, because of the fact that the carbamate ion has instability issues, due to its large steric hindrance [29]. Hence, the formation of a smaller molecule is favored, such as a hydrogen carbonate ion, as proposed by Kim et al. [28,32] (see Equations (3) and (4)).

(3) $$CO_{2}+H_{2}O⇌H_{2}CO_{3},$$

(4) $$H_{2}CO_{3}+R_{1}-NH-R_{2}⇌HCO_{3}^{-}+R_{1}-NH_{2}^{+}-R_{2}.$$

In this case, the overall stoichiometric ratio between carbon dioxide and a given amine moiety is 1:1, theoretically granting a higher performance with respect to the previously presented mechanism. In both reaction schemes, the mechanisms occurring at the upstream side of the film are outlined, which represent the complexing of CO_2_ to the carrier molecule once the gas is dissolved. The opposite reaction will take place at the downstream side, decomplexing the carbon dioxide and allowing the molecule to pass once again to the gaseous phase. 

Historically, the earliest examples of FTMs were supported liquid membranes (SLM), consisting of an immobilized liquid phase, where free carriers could freely move and interact with the target species. These would dissolve on the upstream side and form complexes with the mobile carrier; as a unit, this complex would diffuse through the liquid and decomplex on the downstream side [33]. Another approach to this issue is represented by the use of fixed site carrier (FSC) membranes, where the functional groups are covalently bonded to the matrix polymeric backbone or a dispersed secondary phase and, rather than being free to move through the entire volume of it, their position is limited in the vicinity of an equilibrium point [24,30,34]. Both approaches were explored in research and both showed strengths and weaknesses; small mobile carriers tend to leak and evaporate [35], while fixed carriers can struggle to achieve the same diffusion rates due to lack of mobility. For these reasons, this work focused on the usage of mobile carrier molecules with a high vapor tension and low volatility, combined with a strong presence of aminated functional groups. A category of molecules, which checks all the requisites here laid out, is represented by amino acids and amino-acid salts, which are gaining a certain interest in the field of FTMs [36,37,38,39]. 

For this purpose, l-arginine was selected, since it possesses a strong alkaline polar nature, combined with a high solubility in water (182 g/L [40]) and a relatively low price with respect to other amino acids. As a matrix to hold the arginine and allow the uptake of humidity, carboxymethylated nanocellulose (CMC-NFC) was chosen. Cellulose is the most abundant biopolymer on earth. Extracted most often from wood or annual plants, cellulose fibers are composed of several fibrils which were firstly isolated by Turbak et al. [41] in 1983 and are usually present as microfibrillated cellulose (MFC), cellulose nanofibrils (CNF), or nanofibrillated cellulose (NFC). These kinds of nanocelluloses are some of the most studied bio-based materials and are often reported from production until application in several books and reviews [42,43,44,45]. 

Since 2008, NFC development demonstrated exponential interest from researchers, as well as industrial companies, with more than 60 producers worldwide. More recently, in 2016, NFCs were identified as the second bio-economy priority in Europe thanks to their properties. Indeed, NFCs display high mechanical resistance and excellent barrier level, and they are also biodegradable and biocompatible, highlighting this bio-based material as an excellent candidate in several applications such as packaging [44], paper and board [46], composites [47], printed electronics [48], biomedical devices [49], etc. Also, in the field of membranes, nanocellulose is gaining attention; in the last three years, several papers appeared using this material as a base for the production of gas separation membranes [50,51,52,53].

In order to isolate NFCs from fibers, an enzymatic or chemical pretreatment is performed in order to weaken cellulosic fibers, as well as to reduce the energy consumption of the production process during the mechanical step [54,55,56,57]. One of the chemical pre-treatments applied to cellulosic fibers is carboxymethylation [58,59]. This modification was already reported in numerous papers with specific properties given to carboxymethylated NFC, i.e., highly charged surface (which can lead to ionic interactions with other molecules), and a lot of acid groups at the surface (for post-modification purpose) in comparison to neat NFC [60,61]. Due to its highly polar surface, CMC-NFC fibers were deemed to have a higher chance than plain nanocellulose to positively interact with the amino acid arginine, which is rich in alkaline polar groups. Overall, this combination represents an interesting approach to gas separation membranes and the use of sustainable materials.

Following this line of thought, in the present work, different CMC-NFC/l-arginine composite films were prepared and tested to understand their potential as CO_2_ separation membranes. Permeability tests were performed for CO_2_ and N_2_ at 35 °C and different relative humidity; water vapor sorption experiments were also conducted at the same temperature to relate the permeability to actual water content in the membrane and to better understand its role in the enhancement of membrane performance.

As stated, carboxymethylated NFC can easily react with different molecules due to its amine functional group. In the line of sustainability and green technology development, it appears essential to prepare new membranes for carbon capture using natural molecules, i.e., arginine and bio-based membranes such as CMC-NFC. To the best of our knowledge, there are very few papers and amine-based functional groups in carbon capture membranes, mainly dealing with NFC - aminated polymers blends [50,51] or with neat NFC grafted with aminosilanes [62]. In the present work, CMC-NFC was used as a membrane with arginine as a mobile carrier for CO_2_. 

## 2. Materials and Methods 

### 2.1. Carboxymethylated Nanocellulose Synthesis

Carboxymethylated nanocellulose (CMC-NFC) was kindly provided by INOFIB (Saint-Martin-d’Hères Cedex, France) as a water suspension with a solid content of 1.7 wt.% and a surface charge of 2600 µequiv/mol. 

CMC is usually synthetized by the alkali-catalyzed reaction of cellulose with chloroacetic acid. The protocol used for the modification was based on the one developed by Wågberg et al. [58] 

A total of 180 g of eucalyptus fiber was pretreated. The eucalyptus fibers were first dispersed in water and then solvent-changed to 4 L of ethanol. The fibers were then impregnated with a solution of 175.1 g of monochloroacetic acid in 820 mL of isopropanol corresponding to 10 M OH equivalent with respect to cellulose. A solution of 26.5 g of NaOH in 3.3 L of isopropanol was then added to fibers that were heated to just below boiling temperature in a 10-L reactor fitted with a condenser. This carboxymethylation reaction was allowed to continue for 6 h. Following this carboxymethylation step, the fibers were filtered and washed: first with 20 L of deionized water, then with 3 L of acetic acid (0.1 M), and finally with 15 L of deionized water. The fibers were then impregnated with a 3-L NaHCO_3_ solution (4 wt.% solution) for 60 min in order to convert the carboxyl groups to their sodium form. Finally, the fibers were washed with 15 L of deionized water and drained on a Buchner funnel. In order to obtain CMF-NFC, carboxymethylated fibers were mechanically treated using a Masuko Grinder^®^ device with a speed of 1500 rpm and a gap of −10 µm between the two grinding stones. A gel at 1.7 wt.%. CMC-NFC was obtained after 10 passes.

Morphological studies on CMC-NFC were performed using the FEI-Quanta 200 Scanning Electron Microscope (SEM). The accelerating voltage (Extra High Tension or EHT) was 10 kV for a working distance of 9.7 mm. A drop of diluted CMC-CNF suspension was deposited onto a substrate covered with carbon tape and dried using a vacuum pump and then coated with a layer of Au/Pd (gold/palladium) and an Everhart–Thornley Detector (EDT) was used.

The CMC-NFC gel was characterized by conductimetric titration according to the ISO 638:2008 procedure. The CMC-NFC was acidified to a pH around 2.8 using hydrochloric acid (0.1 M) to convert the carboxyl groups into their acid form (–COOH). The titration was performed with a sodium hydroxide solution (C = 0.05 M). The conductivity of the suspension was measured for each addition of NaOH solution, until pH 10 was reached. Samples were triplicated and the average of the equilibrium concentration was used for calculation of charge.

Infrared spectra were recorded on films on CMC-NFC, using a Perkin-Elmer SP100 spectrometer. For each sample, the diamond crystal of an attenuate total reflectance (ATR) apparatus was used. The torque applied was kept constant to ensure the same pressure on each sample. Triplicates were performed for each sample and the best representative spectra were kept for consideration. All spectra were recorded between 4000 and 600 cm^−1^, with a resolution of 1 cm^−1^ and eight scans.

### 2.2. Membrane Fabrication

A solvent casting protocol, similar to previous works [50,51], was adopted, in order to obtain homogeneous films. l-Arginine (purchased from Sigma-Aldrich with a reported purity >99.5%) was added directly to the CMC-NFC water suspension in powder form and dissolved at room temperature via magnetic stirring for 1 h at 1000 rpm, until no solids could be discerned in the solution. Three different loadings of the amino acid were investigated (15, 30, and 45 wt.%, calculated with respect to the solid content of the suspension) plus a blank sample of pure CMC-NFC.

Due to its high viscosity, during the stirring phase, the suspension tended to incorporate a large amount of air in the form of bubbles. These would eventually result in imperfections in the final film. For this reason, it was centrifuged in mild conditions (10 min, 4500 rpm), in order to separate the gas phase from the liquid one. Subsequently, the homogenized blend was poured into glass petri dishes (11 cm in diameter) and dried in a ventilated oven at 35 °C on a leveled plane for two days. Once dried, the films were peeled off and their thickness was evaluated via a disc micrometer (Mitutoyo, Series 227-221). The thickness was between 35 and 45 µm for the arginine-loaded films and between 15 and 20 µm for the unloaded membrane. An average of three samples were fabricated for each composition.

### 2.3. Water Sorption

**Figure 1:**
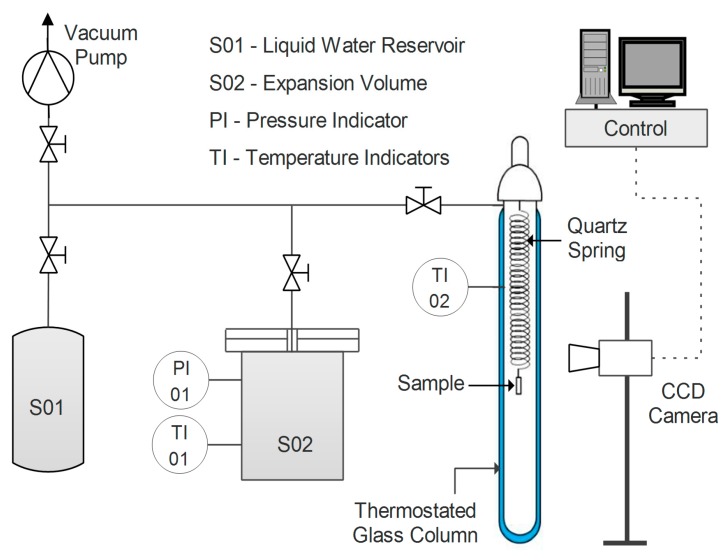
Layout of the quartz spring microbalance used to measure water vapor uptake.

Water uptake was tested for the different samples in film form via a quartz spring microbalance [63]. The basic functioning of this system relies on a quartz spring of known elastic constant, from the bottom of which the sample is hung. Both spring and specimen are enclosed in a glass, thermostatic column, where the pressure of water vapor can be controlled (Figure 1). 

The variation in weight of the sample is recorded by measuring its displacement via a digital charge-coupled device (CCD) camera. The amount of water gained by the sample at each step can be obtained by applying Equation (5).

(5) $${\left[m_{water}\right]}_{i}=\left(h_{i,t\to \infty }-h_{0}\right)\cdot \frac{k}{g},$$

where *h* represents the vertical coordinate of the sample, *k* is the elastic constant of the spring, and *g* is the gravitational acceleration. Moreover, through the analysis of the transitory phase of a sorption step, the diffusion coefficient of water in the film can also be estimated at a given concentration via Equation (6) [64].

(6) $$\frac{m_{sample}}{m_{sample,\;\;t\to \infty }}=1-\underset{n}{\sum }\frac{8}{{\left(2n+1\right)}^{2}\pi^{2}}exp\left[\frac{-D{\left(2n+1\right)}^{2}\pi^{2}t}{L^{2}}\right].$$

The diffusion coefficient *D* is fitted via a graphical interpolation of the experimental data as a function of time *t*, knowing the half-thickness of the sample (*L*). 

All tests were carried out at a temperature of 35 °C and with a water activity ranging from 0.25 to 0.80.

### 2.4. Permeability

**Figure 2:**
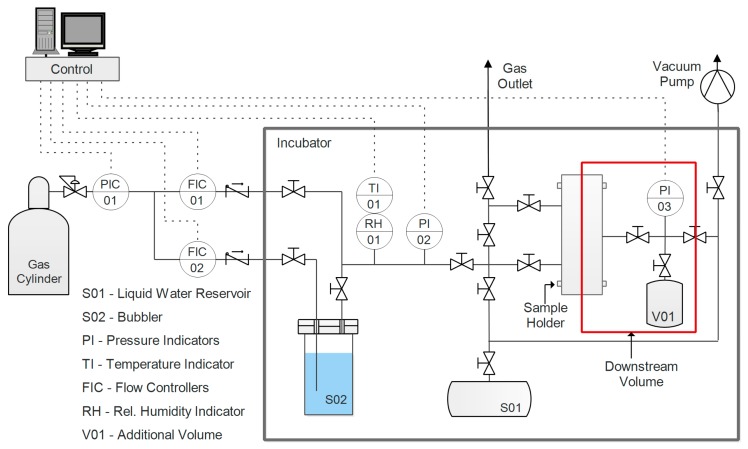
Diagram of the humid single-gas permeation apparatus.

Single-gas permeability in humid conditions of the fabricated films was evaluated for two gases, CO_2_ and N_2_. Before testing, the samples were prepared by cutting them in a circular shape and masking the outer part with aluminum tape sealed with epoxy resin. This was done to avoid direct compression of the film by the rubber O-ring utilized to ensure a good seal in the sample holder. The apparatus used for this purpose, the layout of which is presented in Figure 2, had a set-up incorporating a fixed volume, variable pressure, and a humid permeometer.

A thorough description of the system and its corresponding protocol can be found in a previous work [65]. Pressure (*p*_1_) is constantly measured and recorded in the downstream volume (V). On the upstream side, pressure *p*_2_ is maintained constant, while a flow of humidified gas is fed to the sample. Given these two values and the variation of the downstream pressure over time, Equation (7) can be applied to calculate permeability, assuming an ideal gas state.

(7) $$P=-{\left(\frac{dp_{1}}{dt}\right)}_{t\to \infty }\frac{V}{RT}\frac{L}{A}\frac{1}{\left(p_{1}-p_{2}\right)},$$

where *A* represents the effective area of the membrane, *L* is its thickness, *T* is the absolute temperature, and *R* is the ideal gas constant.

Prior to experiments, the sample and the system were evacuated overnight via a vacuum pump, in order to remove all volatile compounds such as gases absorbed by the film or compounds from the adhesives used in the sample preparation. The following step consisted of introducing pure water vapor to condition the film prior the permeation; pressure was controlled until it reached a stationary value, corresponding to the relative humidity, at which the test was conducted. The gas upstream was then humidified to the same value of the conditioning step, and the test was started by exposing the membrane to the gas. With water in a condition of thermodynamic equilibrium between the two sides of the film, the only contribution to the pressure increment in the downstream volume was eventually represented by the permeation of the incondensable gas (either carbon dioxide or nitrogen, depending on the test). Throughout this work, permeability is expressed in Barrer, and its definition and conversion to International System of Units (SI) units are outlined in Equation (8).

(8) $$1\;\mathrm{Barrer}=10^{-10}\frac{{\mathrm{cm}}^{3}\left(\mathrm{STP}\right)\cdot \mathrm{cm}}{{\mathrm{cm}}^{2}\cdot \mathrm{cmHg}\cdot s}=3.364\cdot 10^{-16}\frac{\mathrm{mol}}{m\cdot \mathrm{Pa}\cdot s}.$$

Gas selectivity was evaluated as the ideal ratio between pure gas permeability, an approximation justified by the low downstream pressure. Tests were performed at 35 °C, with a humidity ranging between 70% and 95%, as lower values were generally characterized by very low permeability.

## 3. Results and Discussion

### 3.1. CMC-NFC Characterization

**Figure 3:**
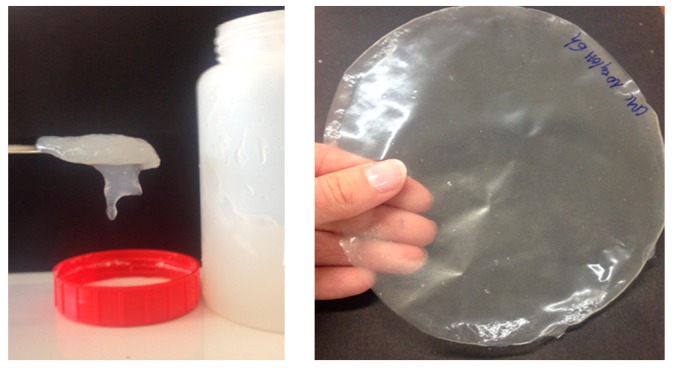
Images of the carboxymethylated nanofibrillated cellulose (CMC-NFC) suspension (**left**) and a sample of the casted film (**right**).

Firstly, a visual observation after the grinding process is shown in Figure 3. This picture clearly shows the gel-like structure and the transparency of the films obtained with the pretreatment done on the fibers.

**Figure 4:**
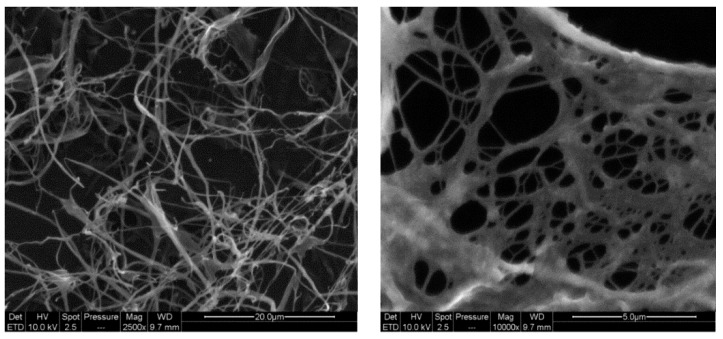
SEM images of a film of carboxymethylated nanocellulose, highlighting the fibrous three-dimensional structure of the material.

Moreover, SEM analyses were performed to point out the nanostructure of CMC-NFC, as represented in Figure 4. 

**Figure 5:**
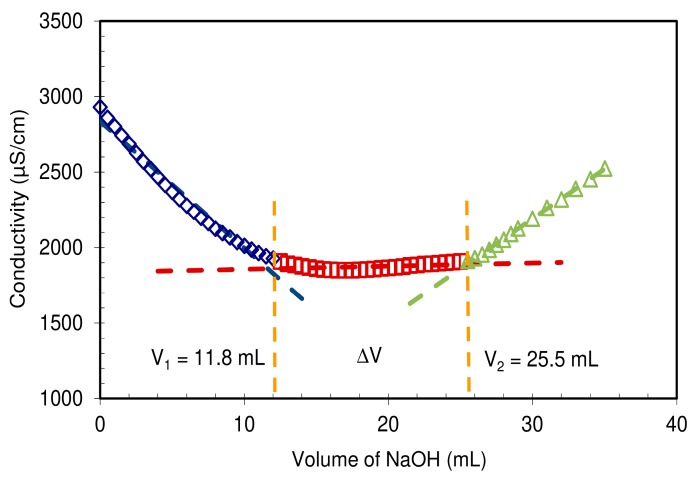
Outcome of conductimetric titration on a CMC-NFC suspension for determining the surface charge of the nanofibers.

The diameter of CMC-NFC was between 80 nm and 150 nm, determined by digital image analysis (ImageJ software) of SEM pictures (a minimum of 50 measurements was performed). Figure 5 presents the quantification of carboxylic content following the titration method from ISO 638:2008.

In comparison to the cellulosic fibers used as raw materials for the preparation of these NFCs, displaying a total charge around 22 µeq·g^−1^ (data not shown) mainly originating from hemicelluloses, Figure 5 clearly shows that CMC-NFC was charged thanks to carboxymethylation pre-treatment yielding the formation of –COOH groups which cause the wide plateau visible in the titration chart. The total charge of CMC-NFC was around 2600 µeq·g^−1^ of carboxylic groups and confirms the occurrence of the modification.

**Figure 6:**
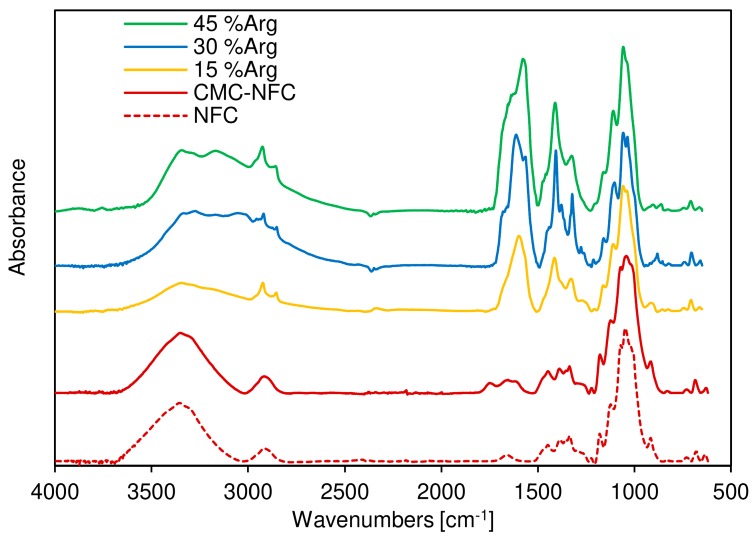
Fourier-transform infrared (FTIR) spectra of plain cellulose, carboxymethylated nanocellulose, and the CMC-NFC/arginine blends.

To ensure this first conclusion, Fourier-transform infrared (FTIR) spectroscopy was also performed as shown in Figure 6, where the FTIR spectra of both the cellulose fiber sample (dotted line) and the CMC-NFC (full line) are presented. They display similar characteristic bands attributed to cellulose substrates. Indeed, the bands around 3496 cm^−1^ (O–H), 1110 cm^−1^ (C–O of secondary alcohol) (used for the normalization of spectra), and 2868 and 2970 cm^−1^ (C–H from –CH_2_–) are present in both samples. 

The main difference between the two spectra was the peak attributed to carboxylic acid vibration around 1745 cm^−1^ due to the larger number of carboxylic groups provided by the carboxymethylation process. A second peak can be also attributed to the ionized form of the carboxylic acid group around 1614 cm^−1^. 

FTIR spectra of the CMC-NFC/arginine blends were also acquired in order to assess the presence of the amino acid within the polymeric matrix. A new peak was first encountered at 3151 cm^−1^, related to the stretching of the N–H bond which extended and partially overlapped with the O–H stretching band. The resulting broad peak suggested the presence of an extensive hydrogen bonding network in the blend. Another significant indication of the growing presence of l-arginine was determined by the sharp peak at 1590 cm^−1^, which was due to the out-of-plane bending of the N–H group, and the series of peaks between 1420 and 1320 cm^−1^ could be related to the symmetrical bending of the CH_3_ group, as suggested by Kumar et al. [66]. 

In conclusion, FTIR analysis confirmed that plain cellulose was successfully modified into carboxymethylated nanocellulose and efficiently blended with different amounts of arginine.

### 3.2. Water Sorption 

**Figure 7:**
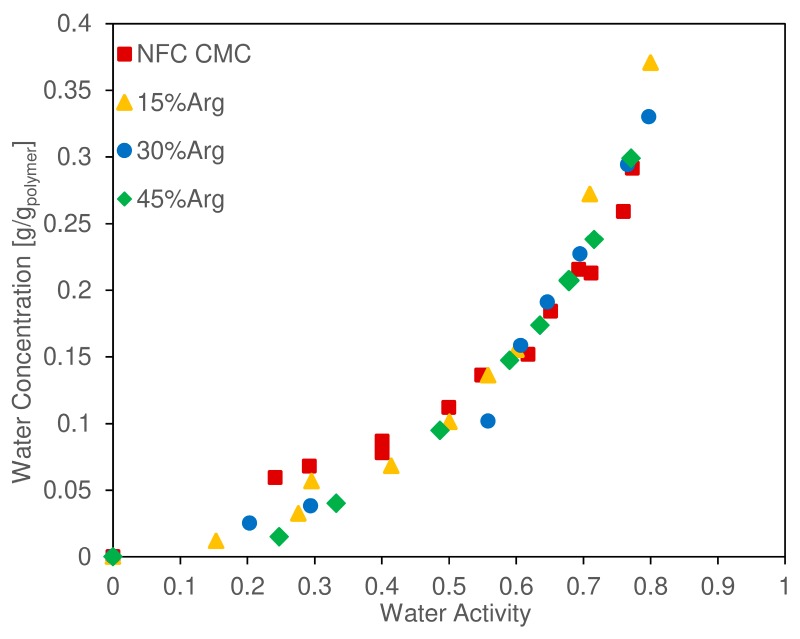
Water uptake of CMC-NFC films with different loadings of arginine at 35 °C.

All the isothermal sorption curves are collected in Figure 7, which presents the water uptake expressed with respect to the dry weight of the film as a function of water activity. From a first view of the data, no significant difference can be outlined when the content of amino acid was incremented in the film. This shows that the water uptake of the nanocellulose/arginine blend does not seem to depend on the ratio between the two components, even if some difference can be observed in the general behavior. In particular, up to an activity of 0.5 and an uptake of about 0.1 g/g_pol_, the water concentration increased with an approximately linear slope with pure NFC films, showing higher water uptake and a positive intercept of the linear trend with respect to the loaded samples. From this point onward, the slope of the isothermal curve changed, acquiring a more exponential trend. Values here ranged between 0.15 g/g_pol_ at an activity of 0.60, up to 0.33 g/g_pol_ at 0.79, and all curves were superimposed with no differences visible among different materials. This dual trend was already observed in previous works on vapor sorption in NFC [50,51], and it was explained by a first phase controlled by the adsorption of water onto the polar moieties of the fibers, followed by a second phase commonly linked to water clustering [67,68] and film swelling, which is also related to the adsorption of a subsequent layer of water molecules onto the first one, exponentially increasing the uptake of water. 

Considering the differences between pure and arginine-loaded NFC, it seems that, at low RH, the pure cellulose nanofibrils adsorb water more easily than composite films, suggesting that, in the latters, some of the acid groups on the surface are less available, due very likely to the interaction with the alkaline part of the amino acid. Interestingly, the difference indeed disappears at higher humidity when the adsorption of subsequent water layers or absorption in the interfibrillar space is considered. 

In general, the absence of any difference among different films with very different loadings of arginine suggests a similar overall water uptake of both NFC fibers and the amino acid, thereby not varying the total equilibrium water concentration while the relative amount of the two changed.

**Figure 8:**
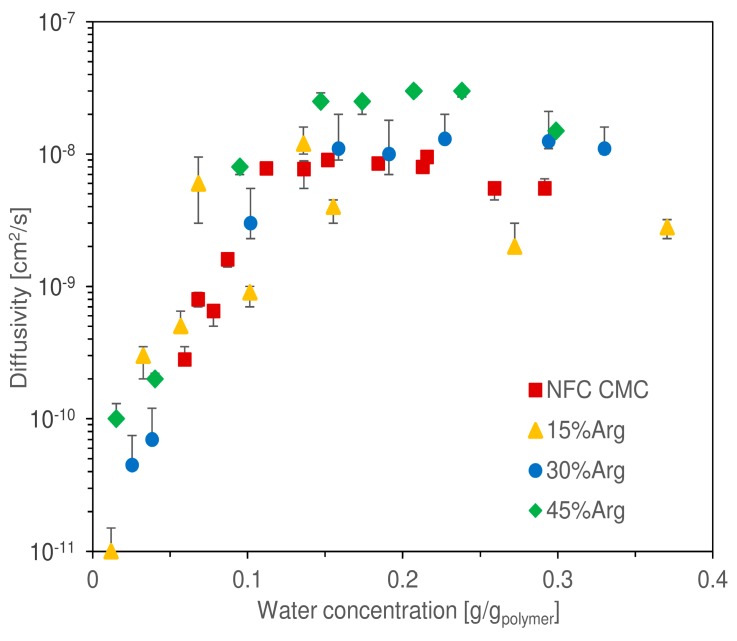
Fickian diffusion coefficient of CMC-NFC films with different loadings of arginine at 35 °C.

Another piece of information acquired from quartz spring balance tests involved an estimation of the diffusion coefficient of water in the hydrated matrix, which is presented in Figure 8 as obtained from the use of Equation (6) on the experimental data of weight change over time.

The reported values represent, therefore, the result of a graphical interpolation, assuming a purely Fickian behavior of the diffusion mechanic. As per the water uptake, it is difficult to discern a significant difference between the curves corresponding to the different loadings of arginine. Again, similarly to that observed in other works regarding nanocellulosic membranes [51,52], diffusivity increased exponentially at low water uptake, specifically between a concentration of 0 and 0.1 g/g_pol_. From the lowest value measured (1.5 × 10^−11^ cm^2^/s at 0.01 g/g_pol_), diffusivity increased by three orders of magnitude reaching 1.2 × 10^−8^ cm^2^/s at 0.13 g/g_pol_. From a concentration of 0.1 g/g_pol_, the exponential trend stopped, and a quite stable plateau was reached for all, with values ranging from 3 × 10^−9^ to 3 × 10^−8^ cm^2^/s (as a reference, the self-diffusion coefficient of water in a liquid state at 35 °C is 2.9 × 10^−5^ cm^2^/s [69]).

In this part of the chart, it could be argued that the different samples tended to reach a plateau at different asymptotic values, for the film with 45 wt.% Arginine occupied the top place at 3 × 10^−8^ cm^2^/s; however, due to the data scattering, high experimental uncertainty was associated with different data. Thus, a final conclusion on the presence of a trend in the asymptotic diffusivity cannot be given without a deeper analysis, which is outside the scope of the present work.

In general, the qualitative trend of diffusivity was justified with the two phases of water sorption, outlined earlier in this section. At low water concentration, the molecules had to diffuse through a tightly packed matrix, determining very low diffusivity values. This process continued until a hydration of about 0.1 g/g_pol_; from this point onward, the nanocellulosic matrix appeared to absorb enough humidity to cause a generalized swelling, which allowed water to diffuse with minimal interaction with the nanocellulosic fibers, thus presenting improved mobility. At the highest water uptakes investigated, diffusivity appeared to show the onset of a downward trend; this would be in accordance with that previously observed for cellulosic fibers by Gouanvé et al. [70].

### 3.3. Permeability 

**Figure 9:**
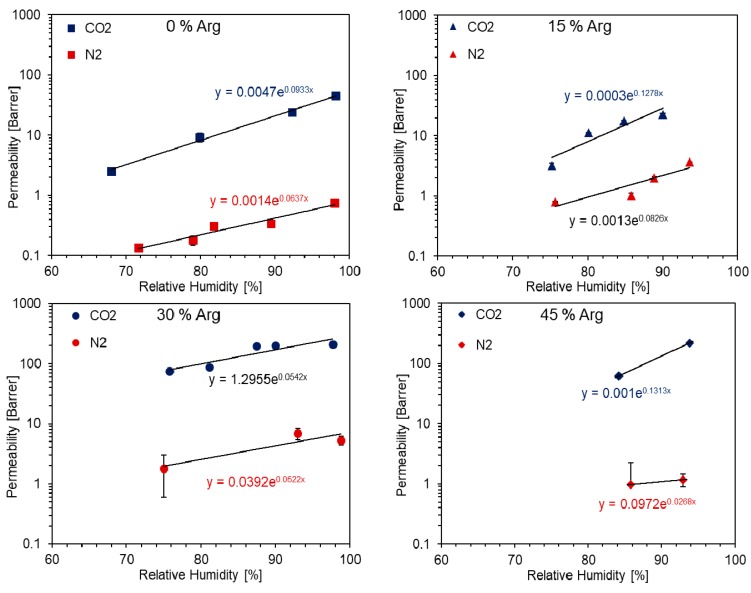
Single-gas permeation results of CMC-NFC with different loadings of arginine with respect to relative humidity.

In Figure 9, the permeation data for CO_2_ and N_2_ are presented, collected for each loading of arginine investigated and expressed as a function of relative humidity. As outlined in previous works [12,50,52,71], the trend of gas permeability with humidity in such hydrophilic materials is an exponential one, with variations of up to 2–3 orders of magnitude going from dry to fully humidified samples. For this reason, the investigation involved a water activity not lower than 0.6, since, below this value, permeability of CO_2_ reached too low fluxes to be interesting for gas separation applications. In the case of the sample with 45% arginine, the investigated RH range was reduced further due to the difficulties working with this material. The very high amount of amino acid indeed caused a loss of mechanical stability in the film, which made it very difficult to obtain reliable data at RH lower than 80%. 

As a reference, pure CMC-NCF showed a CO_2_ permeability ranging from 2.6 Barrer at 68% relative humidity to 45 Barrer at 98.2% RH. These values are in line with that previously observed in similar conditions for unmodified NFC [51]. 

As expected, nitrogen permeability resulted constantly significantly lower than that of carbon dioxide, passing from 0.13 to 0.75 Barrer when RH was increased from 72% to 98%. For the membrane with a content of arginine of 15 wt.%, CO_2_ permeability did not appear to be immediately affected. Indeed, for this membrane, the measured permeability was between 3.2 and 23 Barrer (for a relative humidity range from 75% to 90%). As a consequence, CO_2_ permeation did not display a significant variation regardless of the RH in comparison to the pure NFC reference. N_2_ permeation, instead, resulted higher than pure CMC-NFC, with values from 0.8 to 3.6 Barrer, thus causing a general decrease in the separation performance of the material.

Interestingly, membranes containing a higher amount of arginine showed a different behavior with respect to previous ones. CO_2_ permeability indeed presented a significant increment, with respect to that measured for the non-loaded film with a lower influence of RH. Indeed, for 30 wt.% Arginine loading, values ranged from 75 Barrer at 76% RH to 208 Barrer at 98% RH, while, at 45 wt.%, a CO_2_ permeability of 62 Barrer was measured at 84% RH, reaching a value of 220 Barrer when RH was increased to 94%.

In terms of N_2_ permeation, values obtained at high arginine loading were also higher than those observed for pure CMC-NFC. The increase was, however, lower than in the previous case, thus leading to an overall increase in the membrane separation performance, as better explained in the following section.

Considering that CO_2_ permeability increased at high arginine loading, it is interesting to note that it happened without changing the overall water content in the matrix. This is very different to what was, for example, noted in a previous work [50], where the increment observed when a hydrophilic, aminated polymer was added to NFC resulted indeed very closely related to the increased water uptake of the material. In that case, therefore, the higher swelling induced by water seemed to be the main reason for the improved separation performance, leading to the secondary effect of facilitated transport. Following this thought, the present outcome could be seen as a significant indication (but not definitive proof) of the presence of facilitated transport within the membranes tested.

### 3.4. Selectivity 

Ideal selectivity was calculated as the ratio between permeability of CO_2_ and N_2_ measured at the same relative humidity. Since RH values differed slightly between points taken for the two gases, an interpolation was adopted. Standard humidity levels were fixed, and the respective permeability values were calculated assuming an exponential trend (P=Aexp(B RH)), which for sake of clarity is reported in Figure 9 together with the equation used for calculations.

**Figure 10:**
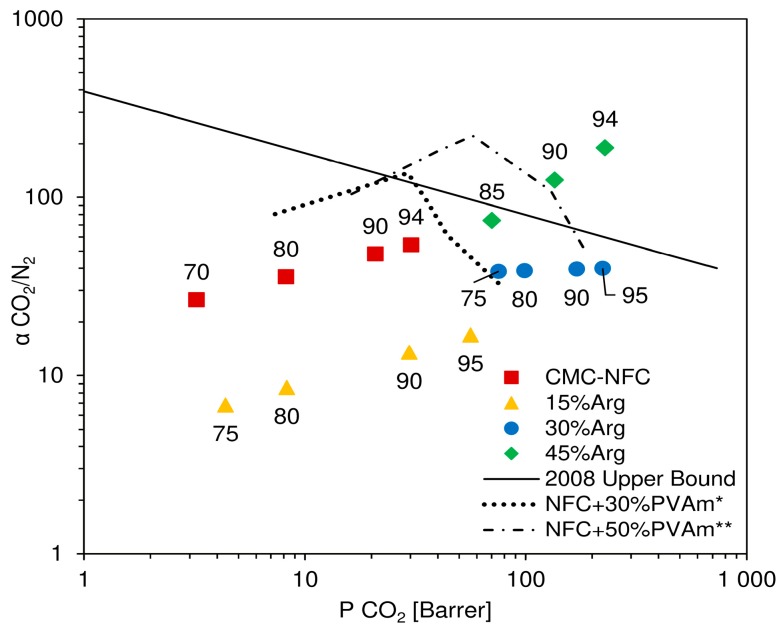
Ideal selectivity of NFC with various contents of l-arginine compared to Robeson’s upper bound and NFC/Polyvinylamine blends from previous works [50,51].

In Figure 10, results of the described procedure are reported in a Robeson plot, where experimental data are presented together with the indication of the relative humidity considered for calculation. In the chart, CMC-NFC clearly showed an incremental trend of ideal selectivity, along with permeability, which was also observed in a previous work [52] for a similar material. In particular, selectivity values ranged from 25 to 56, which represented good separation factors, but still coupled with relatively low permeabilities. Once a small amount of amino acid was introduced (15 wt.%), selectivity values appeared to decrease quite significantly, varying from 4–10 as already expected from the analysis of CO_2_ and N_2_ selectivity variation. 

Once the concentration of l-arginine reached 30 wt.%, selectivity was calculated to range between 30 and 40, without a notable trend with either CO_2_ permeability or relative humidity. These values were quite close to pure NFC but coupled with a significantly higher carbon dioxide permeability (maxed at over 200 Barrer), determining an overall improvement with respect to the base material. Finally, the highest content of amino acid (45 wt.%) determined a quite significant increment in selectivity of CO_2_ with respect to N_2_, showing values from 73 to over 180. In particular, this last point, achieved at very high relative humidity (94 RH%), showed both high permeability and selectivity and placed itself well above Robeson’s upper bound [10], confirming the material’s potential as a membrane for CO_2_ purification.

As it can also be inferred from the permeation data, the increment in selectivity measured for high loadings of arginine appeared to be mostly related to an improved CO_2_ permeability. The fluxes of this gas indeed increased more than the ones of N_2_ at all degrees of humidity investigated, leading to a separation factor continuously improving with water content in the membrane. Interestingly, the present behavior is once again in contrast to that observed when coupling nanocellulose with polyvinyl amine. In that case, the data, also reported in Figure 10 for the sake of completeness, showed a well-defined maximum in selectivity at intermediate RH, before decreasing to values rather close to those which could be calculated for a theoretical water membrane. Also, in this case, the different behavior strongly suggests the existence of facilitated CO_2_ transport across the membrane, which dominates permeability changes in the whole RH range inspected. 

## 4. Conclusions

Carboxymethylated nanocellulose was synthesized starting from natural fibers and was successfully blended with different quantities of the amino acid l-arginine, acting as a mobile carrier for the transport facilitation of carbon dioxide.

Several self-standing films were casted from the blended suspensions and tested for water sorption and gas permeability in humid conditions.

The addition of l-arginine greatly improved both the permeability and ideal selectivity with respect to a pure carboxymethyl nanocellulose matrix. CO_2_ permeability increased seven-fold compared to the base material, from 29 to 225 Barrer, and selectivity with respect to N_2_ grew from 55 to 187 when the matrix was loaded with 45 wt.% amino acid.

Moreover, water uptake experiments allowed indicating that the increment in transport properties was not related to the higher concentration of water in the swollen film, as water solubility was not substantially affected by arginine loading. This is a strong indication, but not definitive proof, of the presence of a facilitated transport mechanism, catalyzed by the presence of the mobile carrier.

While further work is needed to study the stability and durability of the materials, the present results are surely of interest and confirm the high potential of nanocellulose-based facilitated transport membranes for carbon capture application.
